# Surgical repair of a giant congenital left atrial aneurysm presenting with respiratory distress in a neonate

**DOI:** 10.1002/ccr3.4164

**Published:** 2021-05-06

**Authors:** Alwaleed Al‐Dairy, Hazem Aljasem, Samir Srour

**Affiliations:** ^1^ Cardiac Surgery at Faculty of Medicine Damascus University Damascus Syria; ^2^ Damascus University Cardiac Surgery Hospital Damascus Syria; ^3^ PEDIATRIC Cardiology at Faculty of Medicine Damascus University Damascus Syria

**Keywords:** congenital heart disease, left atrial aneurysm, neonatal cardiac surgery, respiratory distress

## Abstract

The presentation of congenital left atrial aneurysm is extremely rare in neonates. The neonate may suffer from severe respiratory distress symptoms, and by then, early surgical management is lifesaving.

## Graphical abstract

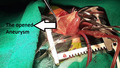

## 1 INTRODUCTION

Congenital left atrial aneurysm is a very rare congenital cardiac anomaly, and very few cases have been reported with neonatal presentation. Even in asymptomatic cases, prompt surgical intervention is indicated to prevent fatal thromboembolic events such as stroke. In neonates and infants presenting early in life, surgical intervention is urgent to relieve respiratory distress symptoms. Herein, we introduce the case of a 3‐week‐old neonate who presented with severe respiratory distress symptoms. Diagnosis of congenital left atrial aneurysm was established and confirmed by transthoracic echocardiography and computed tomography angiography. The patient underwent surgical resection of the aneurysm using cardiopulmonary bypass and cardiac arrest. The patient was discharged from the hospital after 3 weeks with normal echocardiography.

Congenital left atrial aneurysm (CLAA) is an extremely rare congenital cardiac anomaly with potential serious complications on long‐term basis such as arrhythmias, thromboembolic events, and myocardial dysfunction.^1^, ^2^ The first description of CLAA was introduced by Semans and Taussig in 1938.^3^ Growth of CLAA may be attributed to dysplasia of the pectinate muscles which leads to poor myocardial contractility of the left atrium (LA), with resultant progressive LA dilatation.^2^, ^4^, ^5^ It is usually an isolated lesion, and the most common associated lesion is secondary mitral valve regurgitation (MR).^1^, ^2^ The diagnostic modalities of CLAA consist of noninvasive imaging such as transthoracic or transesophageal echocardiography (TTE or TEE), computed tomography angiography(CTA), and magnetic resonance imaging (MRI).^1^, ^3^ Despite the congenital origin of this condition, it may remain asymptomatic and patients may not present until their third decade of life, with very few reported cases of neonatal presentation.^2^, ^4^ Even in asymptomatic cases, prompt surgical intervention is indicated, for the prevention of fatal thromboembolic events such as stroke.^1^, ^2^, ^6^ In neonates and infants presenting early in life, surgical intervention is urgent to relieve respiratory distress symptoms.^7^ Herein, we present a case of a 3‐week‐old neonate who presented with respiratory distress and was diagnosed to have a giant CLAA. Ethical approval for this study was obtained from ethics committee at Damascus University.

## 2 CASE PRESENTATION

**FIGURE 1:**
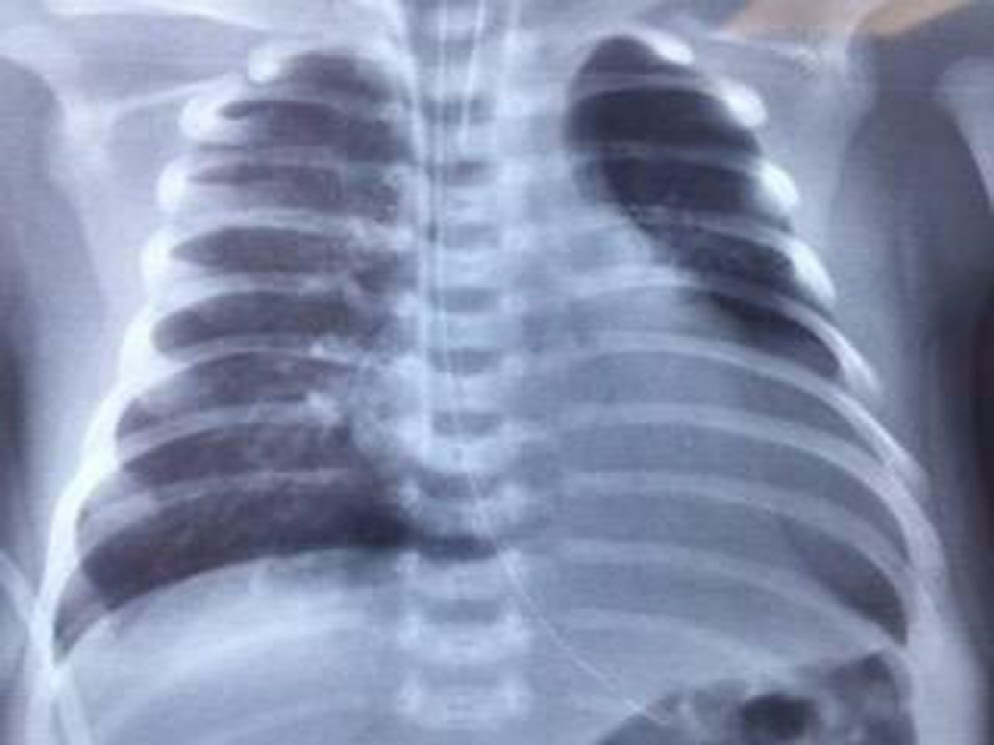
Preoperative CXR

**FIGURE 2:**
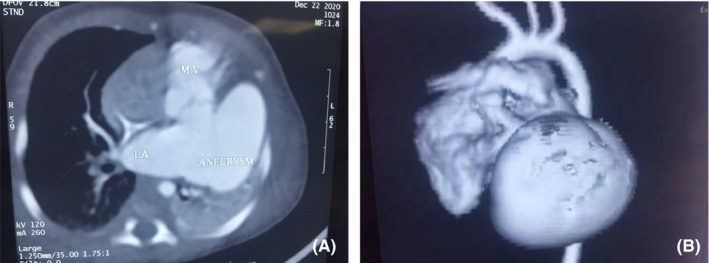
Preoperative CT scan

**FIGURE 3:**
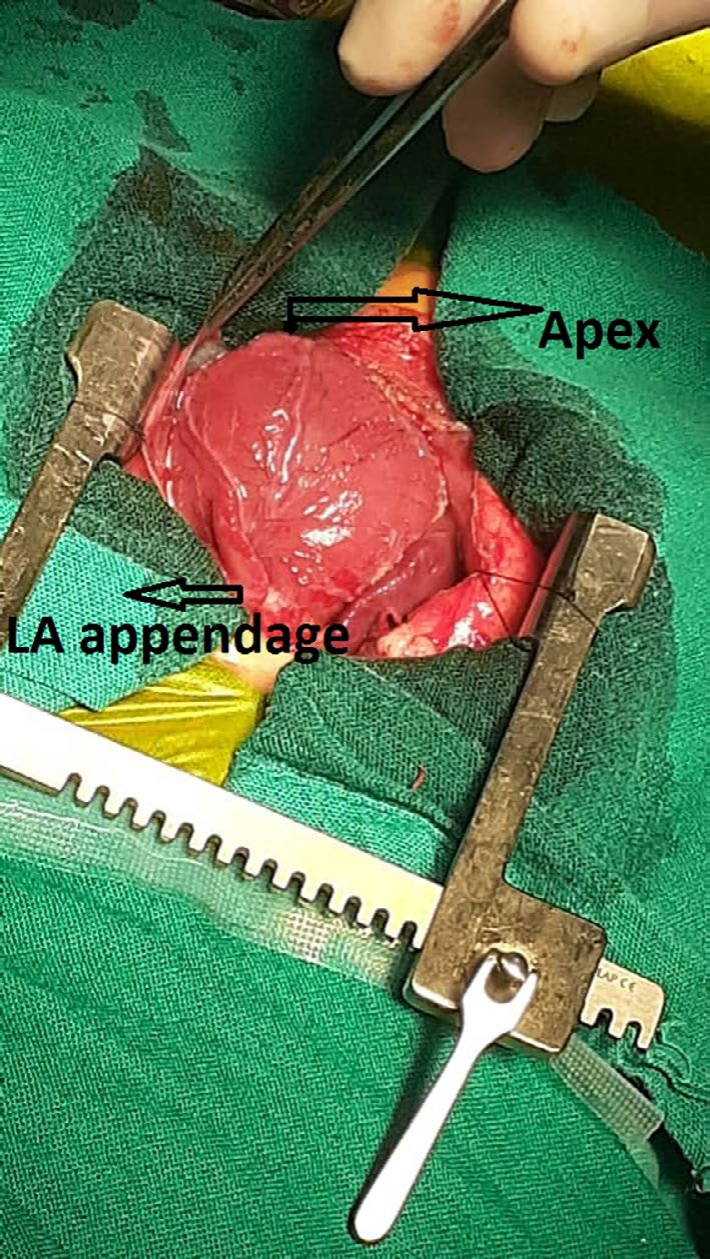
Intraoperative image showing the protruded heart outside the chest and the (intact) LA appendage

**FIGURE 4:**
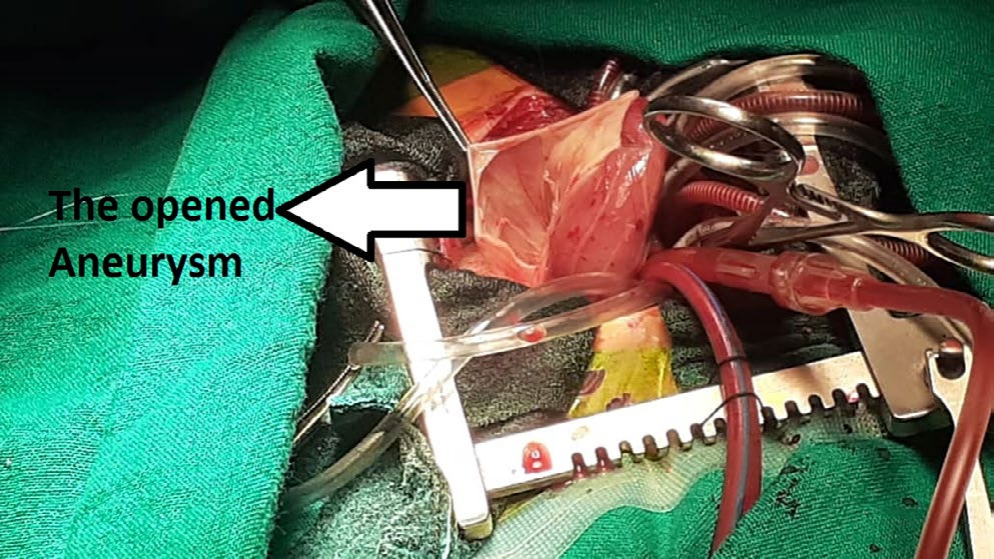
Intraoperative image showing the opened aneurysm

**FIGURE 5:**
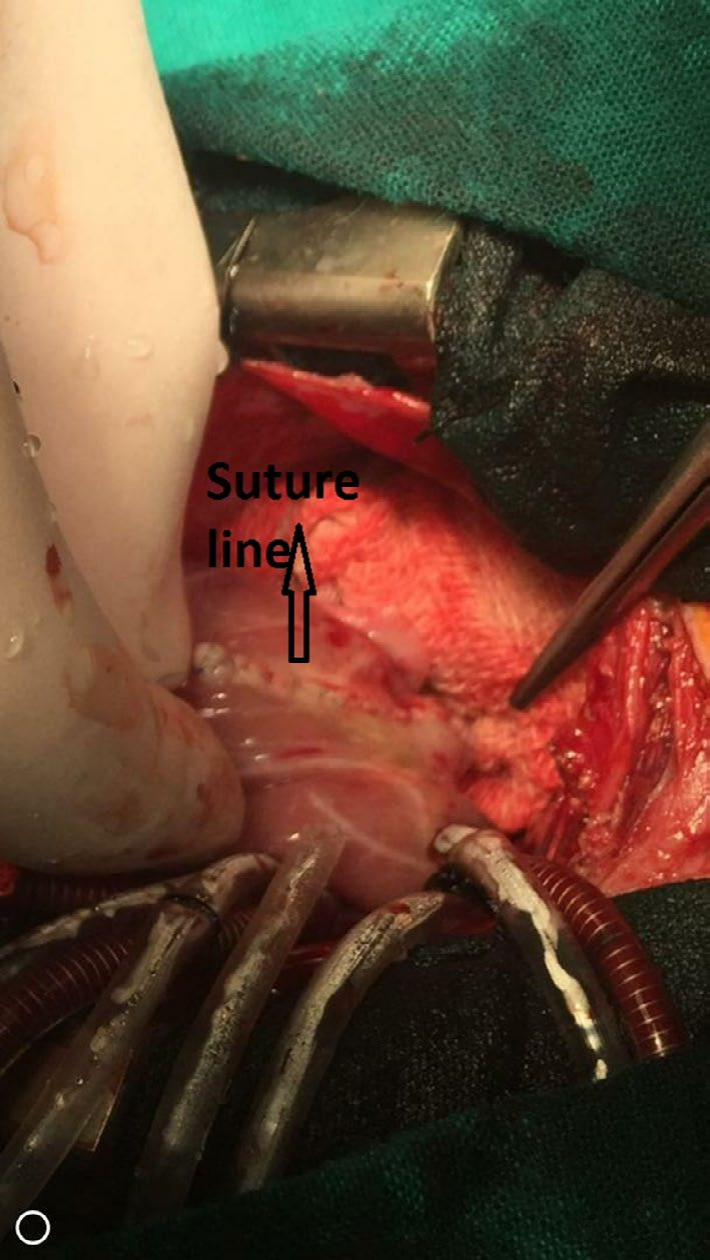
Intraoperative image showing the suture line of closing the defect after resection of the aneurysm

A 3‐week‐old neonate weighing 3400 g was brought to the emergency department of a pediatric hospital. The neonate has recently suffered from severe respiratory distress symptoms and was admitted and promptly intubated. Chest X‐ray (CXR) showed a well‐delineated (mass) on the left border of the heart extending to the left hemithorax compressing the left inferior pulmonary lobe (Figure 1). TTE revealed massive LA aneurysm (about 8 cm) with moderate‐to‐severe MR. CTA was performed to precisely evaluate the LA aneurysm (Figure 2A,B). Depending on the clinical status of the patient, surgical intervention was scheduled on urgent basis. Median sternotomy approach was used. Upon opening the pericardium, the heart protruded outside the chest, and the LA appendage was apparent and intact (it was not part of the aneurysm) (Figure 3). Total cardiopulmonary bypass (CPB) with bicaval cannulation was prepared, and the heart was arrested by antegrade cold blood cardioplegia. The heart was lifted outside the pericardium and rightward (as in the repair of total anomalous pulmonary venous connection), and the aneurysm was found posteriorly and on the left side with very thin wall. The aneurysm was opened, and its communication with LA was confirmed (Figure 4). The left atrial appendage was not the origin of the aneurysm; however, it was originating from the posterior wall of the LA near the posterior mitral annulus. The aneurysm was of sessile nature and extended to the posterior wall of LV adjacent to marginal arteries. It was completely resected, with special attention to avoid injury to the mitral annulus or any of the marginal arteries. The resulted defect was closed by two layers of running 7/0 prolene suturing (Figure 5). The LA was opened, and the mitral valve was inspected. The leaflets and subvalvar apparatus were normal. The valve was tested by saline test and was completely competent. Aortic cross clamp was released, and weaning off CPB was uneventful. The patient was discharged from the hospital after 21 days with significant clinical improvement and normal echocardiography.

## 3 DISCUSSION

Congenital left atrial aneurysm is a very rare entity in neonates with 70% of cases being left atrial appendage aneurysms, and the remainder are left atrial aneurysms.^1^ In neonates and infants, the early presentations of the lesion may be attributed to the secondary MR and airway obstruction, and the risk of complications increases with increase in its size.^8^ To the best of our knowledge, there are only four reported cases of neonatal surgical management of CLAA (one was LA aneurysm, and three were LA appendage aneurysms). Our case represents the smallest age at which CLAA was surgically managed, and it is the largest LA aneurysm presenting in a neonate. Moreover, it had a sessile nature and extended to the posterior wall of LV adjacent to marginal arteries.

## 4 CONCLUSION

Neonatal presentation of CLAA is extremely rare. Early surgical management is lifesaving in neonates with severe respiratory distress symptoms. Surgery for CLAA is safe and uncomplicated, with complete regression of the symptoms even in neonates.

## CONFLICT OF INTEREST

None declared.

## AUTHOR CONTRIBUTION

AA‐D: planned and performed the work leading to the report and wrote and reviewed successive versions and participated in their revisions. HA: wrote and reviewed the successive versions and participated in their revisions. SS: participated in writing the report and approved the final version.

## ETHICAL APPROVAL

The manuscript was approved by ethics committee at Damascus University.

## Data Availability

The data that support the findings of this study are available from the corresponding author, [A.A], upon reasonable request.
